# Are We Modular Lying Cues Detectors? The Answer Is “Yes, Sometimes”.

**DOI:** 10.1371/journal.pone.0136418

**Published:** 2015-09-08

**Authors:** Mathieu Arminjon, Amer Chamseddine, Vladimir Kopta, Aleksandar Paunović, Christine Mohr

**Affiliations:** 1 Department of Psychiatry, University of Geneva, Agalma Foundation, Geneva, Switzerland; 2 Dependable Systems Laboratory, Ecole Polytechnique Fédérale de Lausanne (EPFL), 1015, Lausanne, Switzerland; 3 Laboratory of Electrical and Electronic Engineering, Ecole Polytechnique Fédérale de Lausanne (EPFL), 1015, Lausanne, Switzerland; 4 School of Computer and Communication Sciences (I&C), Ecole Polytechnique Fédérale de Lausanne (EPFL), 1015, Lausanne, Switzerland; 5 Faculty of Social and Political Sciences, Institute of Psychology, Bâtiment Geopolis, Quartier Mouline, 1015, Lausanne, Switzerland; University of Udine, ITALY

## Abstract

We quickly form first impressions about newly encountered people guiding our subsequent behaviour (approach, avoidance). Such instant judgments might be innate and automatic, being performed unconsciously and independently to other cognitive processes. Lying detection might be subject to such a modular process. Unfortunately, numerous studies highlighted problems with lying detection paradigms such as high error rates and learning effects. Additionally, humans should be motivated doing both detecting others’ lies and disguising own lies. Disguising own lies might even be more challenging than detecting other people’s lies. Thus, when trying to disguise cheating behaviour, liars might display a mixture of disguising (fake) trust cues and uncontrolled lying cues making the interpretation of the expression difficult (perceivers are guessing). In two consecutive online studies, we tested whether seeing an increasing amount (range 0–4) of lying cues (LC) and non-lying cues (NLC) on a standard face results in enhanced guessing behaviour (studies 1 and 2) and that enhanced guessing is accompanied by slower responding (study 2). Results showed that pronounced guessing and slowest responding occurred for faces with an intermediate number and not with the highest number of LC and NLC. In particular, LC were more important than NLC to uncertain lying decisions. Thus, only a few LC may interfere with automatic processing of lying detection (irrespective of NLC), probably because too little lying cue information is yet available.

## Introduction

Daily, we meet known and unknown people in various situations. If unknown, we very quickly establish a first impression, *e*.*g*. whether we find the person attractive or like this person. Beyond such superficial impression formations, we even judge upon the character of a new person such as her/his leadership abilities or trustworthiness [1,2]. Most of the time, we take such decisions without having had many opportunities to verify our intuitive impression formations such as having gathered or obtained explicit information on this person’s conscientiousness or values. During first interpersonal encounters, we frequently have little to base our decisions on, apart from first impressions based on verbal and non-verbal cues such as voice, body postures, body movements and facial expressions [3,4,5]. Given that these first impressions are so immediate and surprisingly persistent, various scholars suggested that humans might have developed first impression processing strategies that help deciding very quickly on fitness-relevant perils and benefits when interacting with conspecifics [6]. This latter argument, favored by evolutionary psychologists, refers to cognitive processes explaining humans’ intuitive interpersonal decision making, which can be correct or erroneous, but would have at its ultimate goal an enhanced fitness for survival of both the individual and its group [7,8,9].

A common assumption to such theories in evolutionary psychology is that we have developed survival-enhancing strategies that should benefit mating decisions (*e*.*g*. Is the possible mating partner healthy?) and social investment (*e*.*g*. Does this person follow social norms, can I trust this person, is this person a threat?). Most relevant to the present study is the social investment argument. Evolution might have equipped us with a processing system that quickly signals us whether co-species are likely to be trustworthy or cheaters, the latter better being avoided [8,9,10]. In line with this hypothesis, humans both have an attentional bias for [11] and remember particularly well [12] non-cooperative individuals. Most importantly, humans seem to make lying decisions instantly without much controlled cognitive effort [6,13]. Verplaetse et al. [5] found that individuals decide whether a presented face is lying or not after an exposure duration of as little as 10 seconds. Todorov et al. [2] even suggested that it needs only 33ms exposure duration. Such immediate interpersonal judgments might be based on “modular” processes, *i*.*e*. unconscious, domain specific, fast, automatic, and encapsulated mechanisms [10,14]. Modular processes would allow the quick perception and processing of deception cues, especially microfacial expressions betraying liars’ cheating attempts [5,15,16].

Findings from such studies in evolutionary psychology indicate that individuals are efficient detectors of both trustworthy and cheating cues, potentially due to modular processes. Results from independent studies would, however, contradict these conclusions. For instance, it has been shown that the ability to detect a true deceiver is relatively low [17], ranging from 40% and 60% accurate decisions [18]. Also, individuals were found to need at least 30 minutes interaction time before being able to predict another person’s behavior and cheating in a prisoner’s dilemma game [19]. In addition, trained as compared to untrained individuals had superior lying detection capacities [20]. Given that untrained persons are bad lying detectors (see also, [18]), these authors found that both individuals working in the secret service and clinical psychologists attained about 60%-80% accurate lying judgments, whilst untrained controls performed below chance level. Finally, a recent study showed that justified trustworthiness judgements increase from adolescence to early adulthood [21]. In sum, these studies indicate that lying detection performance is disappointing, *i*.*e*. detection is low, slow and benefits from training effects contrasting assumptions on a fast and efficient modular lying detection system.

Before rejecting the idea of modular processing systems for survival-relevant lying cues, we note various reasons that might explain why lying detection has been disappointing in some of these previous studies. For instance, the experimental material was uncontrolled concerning the role of various possible lying cues (*e*.*g*. body, voice, and speech) [22]. Others argued that deceivers were not really motivated to deceive others [20,4,18] or that deceivers and observers were not interacting with each other [23]. Most important to the present study, liars might not have exhibited clear cheating cues [4]. Indeed, if cheaters would exhibit clear cheating cues, they would be easily recognized and be eradicated very quickly by those who they cheated at [5,23]. Following this line of arguing lying detection might not be a monolithic modular process. Rather, lying detection might rest on two distinct systems. A modular system [10,15,16] by which the observer makes quick judgments based on the analysis of discrete cues, and a non-modular, slower system (see for instance, [24], that might be activated when cheaters do not express clear facial expressions, for instance when liars exibit faked trustworthy behaviors [25,26].

We here investigated the proposition that at least two processes are at stake when people make lying judgments and that these processes result from concurrent conflicting information (lying and non-lying cues). In two independent subsequent online studies, we investigated whether faces, to which we had added an increasing number of lying cues (LC) and non-lying cues (NLC), would be increasingly hard to judge as representing a lying or non-lying face. To give a concrete example, we expected that participants would find it harder to judge whether a face is lying or not when a presented face consists of 4 LC and 4 NLC as compared to a face consisting of 2 LC and 2 NLC. Moreover, we expected that the former decision would take longer than the latter. Ideally, as done in the studies reviewed above, we would present real faces to which a lying history is attached or not. Yet, we do not see the possibility to create real lying and non-lying faces to which we could add a controlled number of LC and NLC. The downside to our approach is that we might measure a propensity to lying judgments and not to actual lying detection (see also [27], but see [28]). Whatever we measure, we consider our results relevant because first impression formation and lying judgements do not necessarily ask for confirmation, but influence behaviour.

In a stimuli selection part, we initially tested lying judgements for previously described LC and NLC [4]. To do so, we asked participants to judge whether presented faces represent somebody lying or not. The presented faces consisted of a series of the same neutral face to which we had added single facial features (*e*.*g*. eye gaze deviated, eye brows raised). The features that resulted in the most pronounced lying and non-lying judgments were selected as LC and NLC. For study 1, we added an increasing amount of these LC and NLC to the same face (range 0–4 LC and 0–4 NLC, respectively). Again, participants judged whether a face was lying or not. In the subsequent study 2, we repeated the procedure of study 1, but enabled the assessment of reaction times when participants decided whether a face is lying or not. Thus, we could test whether individuals were most unsure whether a person is lying when an increasing number of conflicting information (*i*.*e*. LC AND NLC) were added to the same face, and whether this uncertainty would be reflected by enhanced reaction times.

## Stimuli Selection Part: Lying Judgments for Faces Composed of a Varying Number of LC and NLC

### Materials and Methods

#### Participants

Participants were recruited through personal contact by distributing the online link of the study to personally known people (*e*.*g*. family, friends acquaintances) asking them to participate themselves, but also to distribute the online link to further potential participants. This invitation was sent in a bulk email. The addresses were blind-copied. We also posted the online link on our Facebook pages. Thus, participation was entirely anonymous. We were unable to trace back who had actually taken part. The online link remained active for about 7 days. The original sample consisted of 68 participants (age range: 18–68 years, 49 women).

#### Selection of possible LC and NLC in human faces

**Fig 1:**
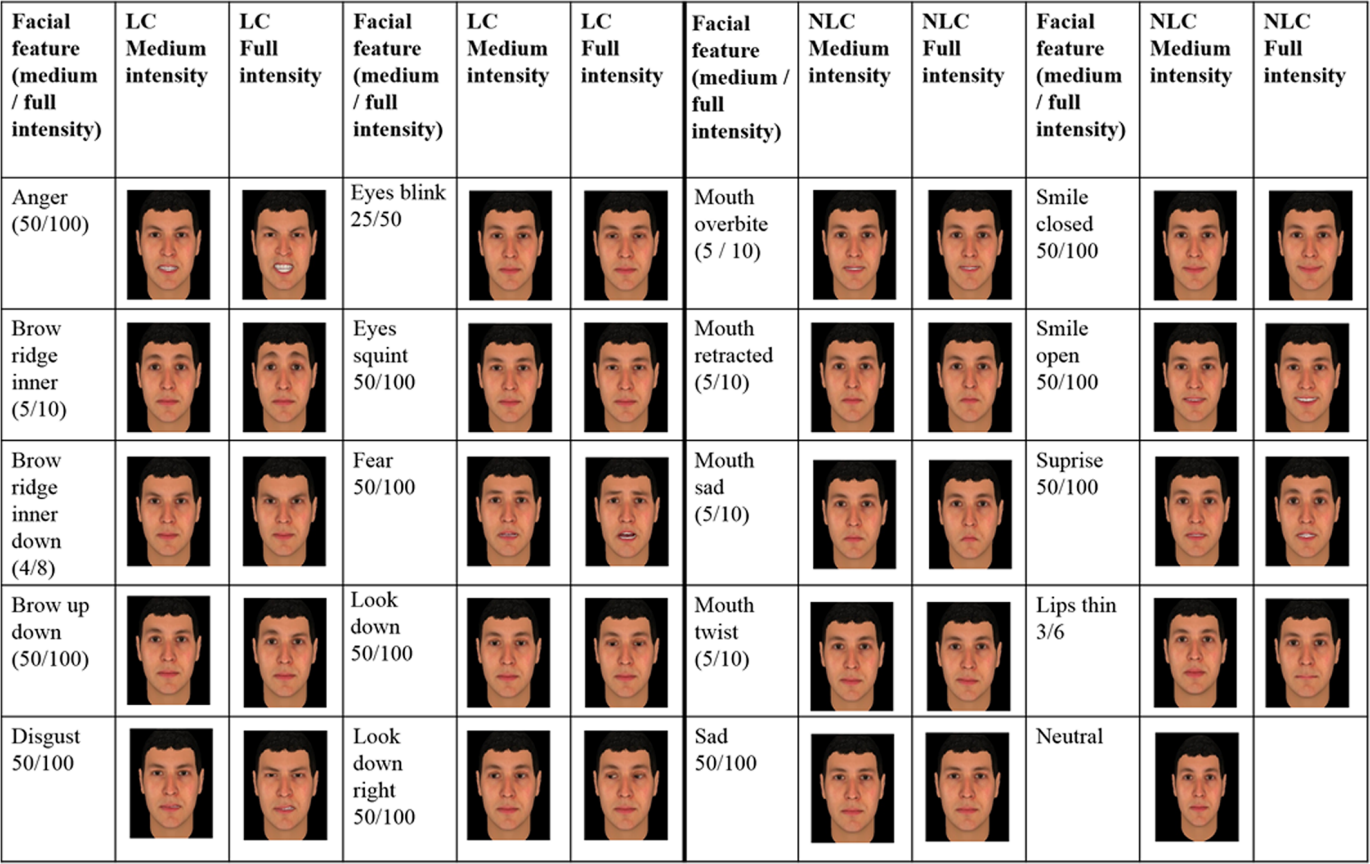
Selection of facial cues. To a neutral face, one potential LC or NLC was added at medium or full intensity using FaceGen Modeller.

DePaulo et al. [4] reviewed the lying literature providing a comprehensive list of possible LC (including face and body, animated as well as static). A priori, we only considered facial features that could be displayed individually on a static face. Using the commercially available FaceGen Modeller 3.5 (http://en.softonic.com/), we added individual facial features of different intensities (*e*.*g*. weak to strong smile, mouth slightly retracted to strongly retracted) to a standard neutral face. Based on the review by DePaulo et al. [4] (see also Fig 1) and what is possible using FaceGen Modeller, we preselected 19 facial cues of which 10 were likely LC and nine were likely NLC. Individually, we added these facial cues to the neutral model face of FaceGen Modeller (see Fig 1). Because intensity might influence to what extent a facial cue is a good LC, we added each facial cue with its full as well as half of its possible intensity. The possible intensities differed between facial cues, *i*.*e*., some ranging in intensity from zero to 10 and others from zero to 100 (Fig 1). Whatever the overall possible range, we always selected the maximum intensity for each facial cue and half of its possible intensity (see Fig 1 for how this applied to the individual facial features).

When adding a facial cue in FaceGen Modeller, this mostly corresponded to one attribute (*e*.*g*. lips thin). In some cases (*e*.*g*. look downwards to the right), however, two attributes had to be added (looking down and looking to the right). Sometimes, we were limited by “shape” properties, *i*.*e*. when we changed one attribute we automatically changed another. Thus, when adding facial cues that were dependent on shape properties, we had to use what the program forced us to do in order to preserve the natural look of the generated face. In practical terms, when we changed the intensity of one facial cue, FaceGen Modeller adjusted another accordingly. All in all, we had 39 pictures including one neutral picture with the remaining 38 pictures consisting of 19 pictures that were presented in two intensities (Fig 1).

#### Procedure

Swiss Law does not require a specific ethic confirmation for these types of study. Yet, studies are required to follow the guidelines of the Helsinki Declaration [29]. When participants activated the online link, they were guided through information screens, on which we provided information on the study, and ethical information in line with the guidelines of Helsinki. In particular, participants were informed on their rights as participants, *e*.*g*. that they could withdraw at any time and that their data would be treated anonymously (only demographic information such as sex, age and education would be recorded). We also informed participants that their continued study participation would be taken as written informed consent including their willingness that we can use their anonymous data for scientific purpose. When participants continued, they received instructions on the actual experiment. For each sequentially presented face, they were informed that they should respond to the question: “is this person lying or not”. If they considered the person to be lying, they were asked to press the F button, otherwise to press the “J” button. After two training trials, participants saw the next slide on which they were reminded that timing would matter, *i*.*e*. to respond as spontaneously and accurately, to leave the left and right index fingers on the “F” and “J” button respectively, and to expect the task to last about 5 minutes. We also displayed a green progress bar on top of the screen. Overall, we presented each face twice with face sequence being randomized between participants. Each face was presented in the centre of the computer screen (face pictures being 624x519 pixels in size) until participants responded. After a short inter-stimulus interval of 300ms, the next picture was presented. For each participant, we recorded lying and non-lying decisions.

#### Data analysis

We screened for participants with potential careless performance, *i*.*e*. unfinished data sets, and participants who might have responded randomly (50% yes responses or 100% responses for the same response key). We accounted for the consistency with which individuals responded for the same pictures (each picture was presented twice). We summed how often individuals responded in the same way for both pictures and divided it by the number of times individuals gave different responses to the picture pairs. Overall, we discarded data from individuals who yielded a consistency value <60% or >95% ensuring that participants responded above chance level without using the same button across all pictures (100% consistency). Because 100% consistency is indeed possible, we verified that to-be-excluded participants had a response bias. This procedure resulted in the omission of 4 participants who yielded a very low (3 women) consistency and 5 participants (4 women) who yielded a very high consistency. Four of these latter participants finished less than 50% of the trials (n = 6). Excluding these 11 participants left us with 57 participants for the final analysis.

For our stimuli selection, we needed distinct LC and NLC. We calculated the proportion with which the 57 participants judged a face to be lying. We aimed to select facial cues that resulted in the most pronounced lying or non-lying judgments. In addition, we had to determine facial cues that could be added individually and in combination to the same face.

#### Stimuli Selection

The proportion with which the 57 participants (41 women, age range 18–68 years) judged the 39 faces to be lying is provided in Table 1. As can be seen, most facial features resulted in 40% to 60% lying judgments. This range is close to 50% lying judgments indicating that the cue is highly ambiguous, *i*.*e*. individuals were not sure whether the facial cue is representing a LC. We did not consider these facial cues further. In addition, percent lying judgments were below 50% for many facial cues (these faces were not considered to be lying). We inferred that very low percentages indicate that the person is perceived of not lying. Finally, some facial cues could not be combined. For instance, it would be impossible to add the facial cue *Smile Open* and the facial cue *Smile Closed* to the same face. Based on these constraints, we selected as LC *Look Down Left* (half scale), *Look Down Left* (full scale), *Lip Thin* (full scale), *Eyes Blink* (full scale). As NLC we selected *Smile Open* (full scale), *Mouth Retracted* (half scale), *Fear* (half scale), *Brows Up/Down* (half scale) (Table 1). Note that *Look Down Left* with full-scale intensity will be counted as adding two LC to the picture.

**Table 1:** Lying judgments for facial cues. Proportion of participants who provided lying judgments for the 38 faces. The facial cue labels are those used in FaceGen Modeller. The final facial cues (used in our subsequent studies) are highlighted in bold for the LC and in italic bold for the NLC.

Cue	Proportion of lying answers	
	Half intensity	Full intensity
Anger	33%	35%
Brow Ridge (Up)	34%	48%
Brow Ridge (Down)	42%	49%
Brows Up/Down	***15%***	40%
Disgust	43%	45%
Eyes Blink	45%	**57%**
Eyes squint	26%	54%
Fear	***23%***	35%
Look Down	52%	53%
Look Down-Left	**76%**	**87%**
Mouth Overbite	38%	52%
Mouth Retracted	***16%***	28%
Mouth Sad	26%	35%
Mouth Twist	30%	53%
Sad	27%	27%
Smile Closed	30%	42%
Smile Open	24%	***22%***
Surprise	25%	35%
Lips Thin	29%	**61%**

We here determined facial cues resulting in the highest proportion of lying and non-lying judgments. Based on a previous review on LC [4] and what we could do with FaceGen Modeller, we had selected 19 facial cues we individually added to a standard neutral face in full and half intensity. Online, a random sample of participants rated for each sequentially presented face, whether the person is lying or not. Results showed that participants were at odds with each other regarding most facial cues, *i*.*e*. they showed a low proportion of lying judgments, or seemed to guess (half of the participants judged the face to be a lying face). For the actual studies, we selected facial cues that were either strong or very weak lying indicators and could be added individually and in combination to the same face.

These facial cues were used to create faces to which an increasing number of LC and NLC were added. These faces are key to test our main study question, *i*.*e*. whether participants as a group would become increasingly unsure as to whether a person is lying or not when exposed to an increasing amount of conflicting information (LC and NLC combined). We added either only facial cues from one category (non-conflicting faces: only LC or only NLC) or facial cues from both categories (conflicting faces). A priori, we assumed that the faces with the highest amount of conflicting information would result in the highest level of uncertainty as to whether the person is lying, *i*.*e*. a proportion of lying judgments around 50%.

## Study 1: Experiment on Lying Judgments for Faces Composed of a Varying Number of LC and NLC

### Materials and Methods

#### Participants

We recruited a new sample of 57 participants (age range: 18–65 years, 29 women). The strategy was the same as in the stimuli selection part.

#### Face decision task

Face stimuli: Having determined the four LC and the four NLC, we again used the FaceGen Modeller software to generate facial stimuli composed of different combinations of the eight facial cues. For each facial category (LC, NLC), we could add 0, 1, 2, 3, or 4 facial cues resulting in 5 x 5 = 25 basic face combination possibilities (see Table 2). For instance, the face combination possibility consisting of 0 NLC and 0 LC would result in one possible face, the standard face. The face combination consisting of 1 LC and 2 NLC would result in 24 possible faces (4 different possibilities to choose 1 among the 4 LC; and 6 different possibilities to choose 2 among the 4 NLC). In the case of 4 LC and 0 NLC (1 possible face), we would obtain the face that displays all LC, and should result unambiguously in a high proportion of lying judgments. In the case of 4 LC and 4 NLC (1 possible face), we would obtain the face of highest conflict. This procedure would result in 256 possible face combinations. To enhance the probability of task completion (short duration) and because we had no reason to assume that our hypothesis required that all possible face combinations were presented, we selected 70 faces from low to high conflict, and presented each face twice (to assess consistency, see also stimuli selection part). In Table 2, we show the frequency with which the 70 faces fell into the different facial cue combination categories (number in brackets). The task procedure was the same as in the stimuli selection part.

**Table 2:** Categorization of faces into conflict groups. We calculated the conflict values for the different facial cue combinations by multiplying the number of LC and NLC. Faces with conflict values of 0 represent the very low conflict group (cells marked with an A), of 1,2, and 3 the low conflict group (cells marked with a B), of 4 and 6 the high conflict group (cells marked with a C) and of 8,9, 12 and 16 the very high conflict group (cells marked with a D). In brackets, we give the distribution of the 70 faces in the different facial cue combination categories (number of faces used in this study / total number of possible faces).

		LC				
	Adding	0 cue	1 cue	2 cues	3 cues	4 cues
NLC	0 cue	0 (1/1)^A^	0 (2/4)^A^	0 (2/6)^A^	0 (2/4)^A^	0 (1/1)^A^
	1 cue	0 (2/4)^A^	1 (6/16)^B^	2 (4/24)^B^	3 (4/16)^B^	4 (2/4)^C^
	2 cues	0 (2/6)^A^	2 (4/24)^B^	4 (6/36)^C^	6 (4/24)^C^	8 (2/6)^D^
	3 cues	0 (2/4)^A^	3 (4/16)^B^	6 (4/24)^C^	9 (6/16)^D^	12 (2/4)^D^
	4 cues	0 (1/1)^A^	4 (2/4)^C^	8 (2/6)^D^	12 (2/4)^D^	16 (1/1)^D^

#### Data analysis

We used the same data cleaning procedure as in the stimuli selection part. This procedure resulted in the omission of 6 out of 57 participants among them five participants (two women) who yielded a very low consistency in performance and one woman who yielded a very high consistency in performance.

To statistically assess whether participants showed enhanced guessing responses for faces of enhanced conflict, we first grouped the 25 possible face combinations into 4 different groups, *i*.*e*. those of very low, low, high, very high conflict. We defined conflict as the value resulting when multiplying the number of LC and the number of NLC (Table 2). Thus, values range from zero (not at all conflicting) to 16 (maximum conflict). Table 2 shows how these conflict values are distributed across the 25 possible face combinations. Using these values, we now partitioned the 25 possible face combinations into four conflict groups with conflict values of zero belonging to the very low conflict group, values of 1, 2, and 3 belonging to the low conflict group, values 4 and 6 to the high conflict group and finally values of 8, 9, 12, and 16 belonging to the very high conflict group (Table 2).

To test whether faces of increasing conflict resulted in more random responses, we calculated a randomness score per conflict group and participant. This randomness score was obtained by dividing the number of lying answers (LA) by the number of non-lying answers (NLA). If the number of LA was smaller than the number of NLA, we divided the number of NLA by the number of LA. Thus, values ranged from 0 to 1 with higher values indicating more pronounced random answering. These values were used for statistical analysis; we performed a one-factor ANOVA with conflict group as within-subject factor on the randomness values. Post-hoc comparisons were performed using paired t-tests. To account for multiple comparisons, we applied a more conservative significance level to our data; we tested at an alpha level of 1% [30]. We applied this 1% criterion, because we were not only interested in reducing the risk of family-wise type I errors, but also in accounting for the increased possibility of committing unwanted family-wise type II errors (e.g. [31,32]). Beyond this decision, for the current study, a more conservative criterion, such as Bonferroni corrections, would be inappropriate [31,32,33]. As reported several decades ago, out of 10 paired comparisons, there is a 40% chance of having at least one significant result when testing at an alpha level of 5% [34]. In our studies, we never performed more than 10 pairwise post-hoc comparisons per ANOVA. Thus, we consider it appropriate to test at an alpha level of 1% [30]. In line with recent recommendations [32,33], we provide effect sizes, here as partial eta-square (PES).

### Results and Short Discussion

**Fig 2:**
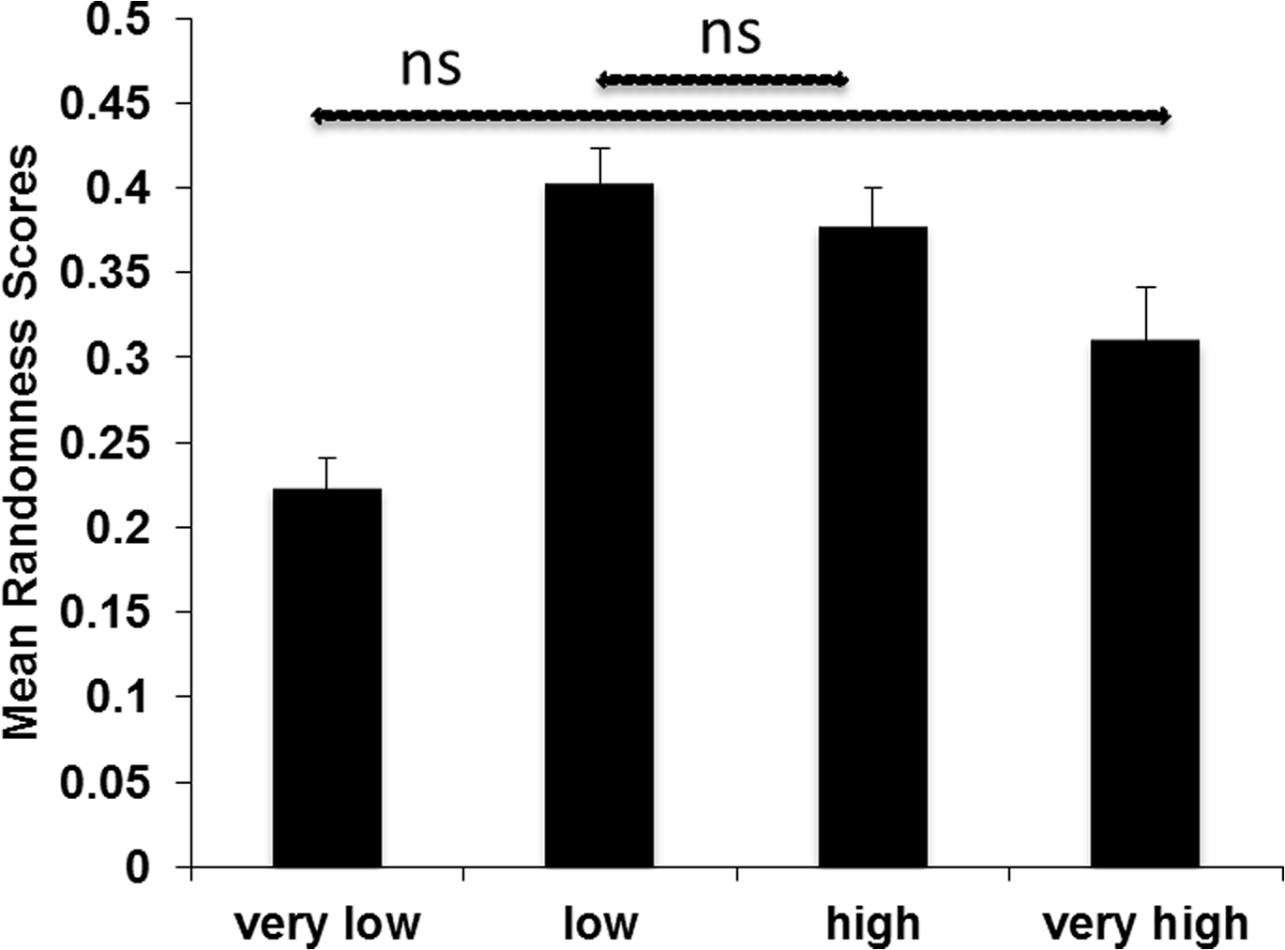
Randomness scores and conflict groups. Mean randomness scores as a function of conflict groups. Vertical bars denote 1 standard error of the mean. If not otherwise stated (non-significant, ns), the pairwise comparisons were significant.

The repeated measures ANOVA on the randomness scores with conflict groups (very low, low, high, very high) as within-subject measure showed a significant main effect, *F*(3, 150) = 15.101, *p*<.001, *PES* =. 23. Post-hoc pairwise comparisons showed that 4 out of the 6 comparisons were significant (all p-values <. 01) apart from the differences between the low conflict group and the high conflict group (p =. 35) and the very low conflict group and the very high conflict group (p = 0.012) (Fig 2). Thus, the lowest randomness scores were observed in the very low conflict group and the very high conflict group. Yet, randomness was not highest in the very high conflict group, but in the low and high conflict groups (Fig 2).

**Fig 3:**
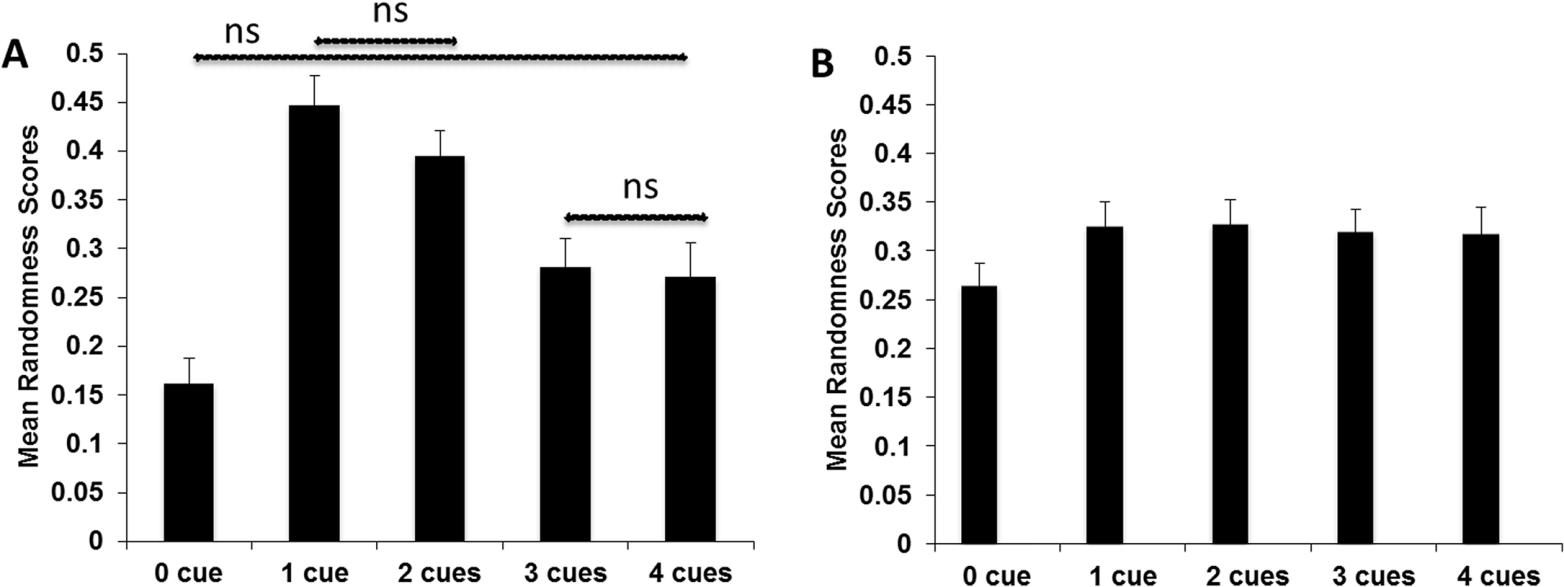
Randomness scores and facial lying cues. Mean randomness scores as a function of an increasing number of LC (A) or NLC (B). Vertical bars denote one standard error of the mean. If not otherwise stated (non-significant, ns), the pairwise comparisons were significant in Fig 3A. Regarding Fig 3B, the main effect was not significant, i.e. the pairwise comparisons were not computed.

Results from this study showed that faces of very low conflict yielded the lowest amount of random responding, *i*.*e*. as a group, people responded rather consistently across these faces. The remaining results were inconsistent with our prediction, though. Participants were not most strongly guessing about the lying intention in a depicted face when seeing faces of the very high conflict group. We predicted the highest probability of random responding for the very high conflict group, because both modular and non-modular systems might be equally activated (see introduction). We observed instead that neutral faces to which an intermediate amount of LC and NLC were added triggered the highest amount of random responding, *i*.*e*. guessing about whether a face is lying or not. In fact, faces of the very high conflict group resulted in lower random responding than the intermediate conflict groups. Given that these findings were unexpected, we further inspected the current data. We calculated randomness scores for the 25 possible face categories separately (Table 3). The values in this table suggest that the influence of the number of NLC is negligible. The highest proportion of random responding occurred when one, two or three LC were added to the neutral face, irrespective of the number of NLC added (bold values in Table 3, see also Fig 3).

**Table 3:** Randomness scores for the face combinations. Mean randomness scores computed across participants for the 25 possible face combinations.

		LC				
	Adding	0 cue	1 cue	2 cues	3 cues	4 cues
NLC	0 cue	.09	**.73**	**.84**	**.37**	.27
	1 cue	.09	**.53**	**.90**	**.60**	.42
	2 cues	.13	**.48**	**.69**	**.56**	.46
	3 cues	.17	**.55**	**.85**	**.57**	.39
	4 cues	.18	**.45**	**.90**	**.44**	.45

To further this observation in Table 3 statistically, we extracted the individual randomness data for the five categories of the two facial cues (LC, NLC), separately. Thus, we performed two separate repeated measure ANOVAs with category (0, 1, 2, 3, 4 cues) as within-subject measure, one on LC and one on NLC. The main effect of category was significant for the ANOVA on LC, F(4,200) = 14.98, p <. 001, *PES* =. 23. Post-hoc pairwise comparisons showed that 7 out of the 10 comparisons were significant (all p-values < 0.01) apart from the differences between faces with 0 LC and 4 LC (p = 0.01), between faces with 1 LC and 2 LC (p =. 17) and between faces with 3 LC and 4 LC (p =. 71) (Fig 3A). The analogue ANOVA on NLC showed no significant main effect, F(4,200) = 1.43, p =. 23, *PES* =. 03 (Fig 3B).

Results from study 1 indicate that increasing conflict (LC and NLC added to a standard face) does not enhance the subjective uncertainty whether a person may be lying or not. An intermediate number of LC resulted in the highest amount of random answering (adding 1 or 2 LC). Moreover, the influence of LC and NLC on lying judgements is not symmetric; *i*.*e*. LC seemed more influential to lying decisions than NLC, because faces with 1 LC as well as 2 LC resulted in the most enhanced random responding, irrespective of how many NLC had been added to these faces. In line with our initial proposition, it may be evolutionarily most advantageous to develop strategies that prevent negative outcomes (being cheated at) as compared to attending to potential positive outcomes [10]. The conditions that resulted in the highest amount of random answering (when 1–2 LC were added to a face) might thus reflect the situation we should be most interested in, *i*.*e*. a lying detection system that rests on our proposed two distinct systems (modular, non-modular). Probably, some non-modular cognitive evaluation might lead to random lying and non-lying decisions across faces and by inference across people.

In study 2, we tested whether the randomness results of study 1 can be replicated and complemented by reaction times for lying judgements. As already pointed out in the introduction, non-modular processing as compared to modular processing should go along with slower responses [24]. Thus, we tested whether random responding, and the inferred uncertainty as to whether a face might be lying or not, is the result of an additional (non-modular) cognitive effort (see again introduction). This additional effort should be reflected by enhanced reaction times [35,36]. To test this possibility, we performed this second study. Thus, we would expect highest randomness scores in the intermediate conflict groups. Moreover, enhanced randomness scores should go along with enhanced reaction times.

## Study 2: Lying Judgments and Their Respective Reaction Times for Faces Composed of a Varying Number of LC and NLC

### Materials and Methods

#### Participants

We recruited a new sample of 68 participants with an age range of 20–44 years (33 women, one person did not indicate his/her sex). We used the same recruitment strategies used in study 1.

#### Face decision task procedure

We used the same faces as the ones used in Study 1 (Table 2). We used the same procedure as the one used in Study 1. New to the current study, we also measured reaction times. In order to assess reaction times in an online study, we preloaded all images into an intermediate memory store before the study begun. This preloading helped us avoiding picture presentation delays due to flaky internet connections. To make sure participants knew the procedure and would be able to respond quickly, we added two practice trials. We also asked participants to give their responses by button press, and not by mouse click. Thus, they were instructed to keep the right and left index finger on the F key and J key, respectively. To enhance timing, we presented a fixation cross for 300 ms before each picture presentation.

#### Data analysis

Three participants were excluded because response consistency was > 95% (2 women, a person who failed to provide the information on sex). These exclusions left us with a sample of 65 participants (31 women, mean age 25.4 years ± 4.11 years).

For statistical analysis, we performed the same analysis on randomness scores as performed in study 1. For reaction times analysis, we first accounted for within and between subject variations in response times. We normalized reaction times for each participant according to the following formula, RT_n_ = (*T—*μ) / σ, where σ is the standard deviation of time, μ is the mean value of response time, and *T* is the absolute response time. Accordingly, larger values represent slower responding. These normalized response times were averaged across faces of the same conflict group. To check for uncommon interruptions in task performance, we individually inspected performance to find particularly long reaction times that could indicate that the participant was interrupted or stopped performing the task for unknown reasons.

The two scores (randomness score, standardized reaction times) were subjected to separate one-factor ANOVAs with conflict groups as repeated measure. Post-hoc comparisons were performed using paired sample t-tests. In line with the previous study, we again tested at a significance level of 1%. Effect sizes are provided by reporting partial eta-square (PES).

### Results and Short Discussion

**Fig 4:**
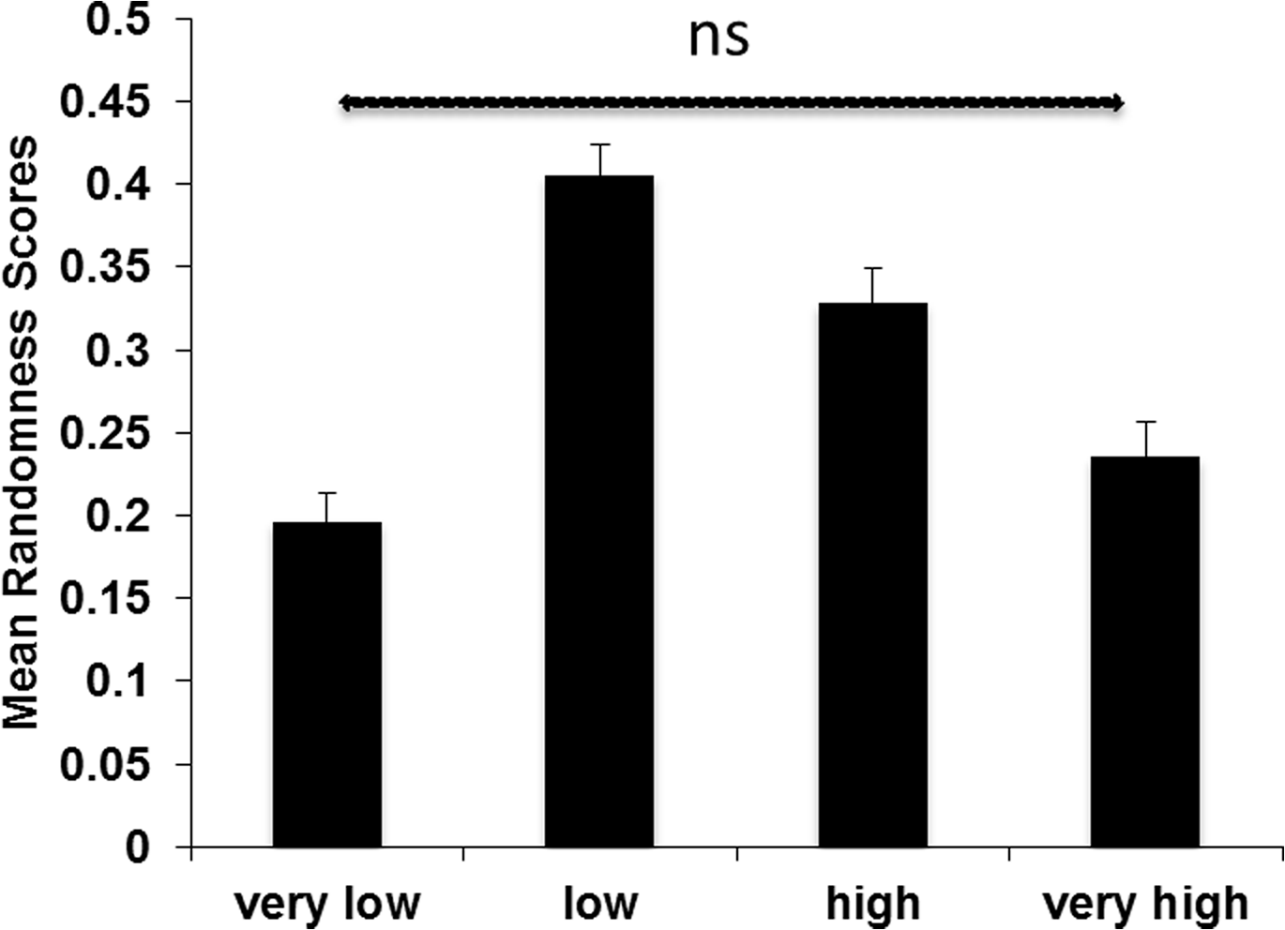
Randomness scores and conflict groups. Mean randomness scores as a function of conflict groups. Vertical bars denote 1 standard error of the mean. If not otherwise stated (non-significant, ns), the pairwise comparisons were significant.

The ANOVA on randomness scores showed a significant main effect of conflict groups, *F*(3, 192) = 32.731, *p*<.001, *PES* =. 34 (Fig 4). Post-hoc pairwise comparisons showed that 5 out of 6 comparisons were significant (all p-values <. 005), apart from a marginal difference between the very low and the very high conflict group (p =. 089). Thus, the intermediate conflict group (in particular the low conflict group) yielded the highest randomness scores (Fig 4).

**Fig 5:**
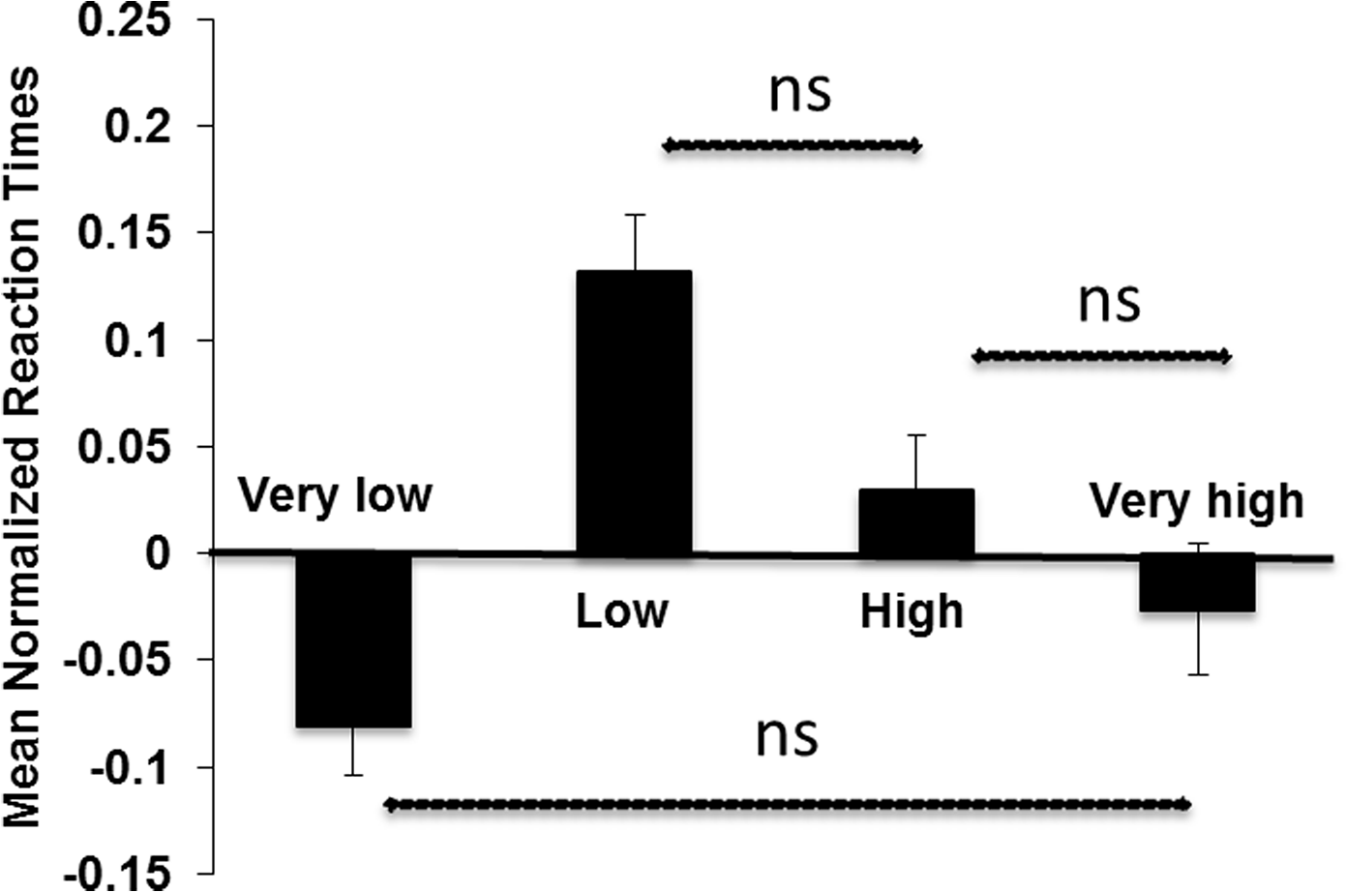
Reaction times and conflict groups. Mean normalized reaction times as a function of conflict groups. Vertical bars denote 1 standard error of the mean. If not otherwise stated (non-significant, ns), the pairwise comparisons were significant.

The second ANOVA on the normalized reaction times showed again a significant main effect, *F*(3, 192) = 9.06, *p* <. 001, *PES* =. 12 (Fig 5). Post-hoc pairwise comparisons showed that 3 out of 6 comparisons were significant (all p-values <. 01), apart from a non-significant difference between the very low and the very high conflict group (p =. 23), between the high and very high conflict group (p =. 18), and between the low and high conflict group (p = 0.017). Thus, reaction times were slowest in the low and high conflict groups (Fig 5).

## General Discussion and Conclusions

This study investigated whether humans might rely on an inbuilt capacity to quickly detect potential liars without having to allocate overt high-level cognitive effort to this goal. Various scholars suggested that efficient cheating detection in humans is based on an unconscious modular system [10,5,15,16]. Others studies questioned such a modular system, because humans are as such bad cheating detectors who will hardly improve in their performance with training [20]. While both sides have supporting data to their views (see also [21]), it seems also possible that modular and non-modular systems are at play when individuals try to detect liars. Individuals do not only need to detect liars (potentially based on a modular system), but they do also know that the liar will try to hide the lying. Thus, individuals might only capture what actually leaks from the liar. In real life situations, liars are likely to aim for neutral facial expressions with some cheating cues nevertheless leaking [25]. Accordingly, we assumed that highly conflicting faces (showing a maximum number of LC and NLC) represent faces that are processed with the highest uncertainty (resulting in the highest amount of random responding across participants), and by inference being processed in a non-modular mode. We assumed that this uncertainty (and non-modular processing) would be reflected in random decisions as to whether a face is lying or not, and an increased reaction time for these decisions [24, 26,27].

We performed two independent studies. In the stimuli selection part, we determined individual LC and NLC. For the actual studies, we created faces with an increasing number of (maximum 4) LC and NLC. Results showed that random decisions were most pronounced for faces to which an intermediate amount of LC had been added. Interestingly, the NLC seemed to contribute little to this effect. In study 2, we replicated this effect. We additionally showed that reaction times were slowest for these intermediate faces. The current results show that our expected behavior (random decisions, slowed responding) was not observed for the most conflicting faces, but for faces to which an intermediate amount of LC had been added.

To discuss the implications of our results we have to account for two major observations. Firstly, random decision taking co-occurring with increased reaction times was not observed for faces of very high conflict. Instead, and secondly, this decision taking occurred for faces to which an intermediate number of LC (*i*.*e*. mainly 1 LC, but also 2 LC) had been added. When we want to consider the first point, we also need to consider the second point. NLC seem to play a minor if not negligible role when making lying decisions on faces. Results showed that random decisions for faces with 1 LC (and partially also for faces with 2 LC) were not further modulated by the presence of 1, 2, 3 or even 4 NLC (study 1). It seems as if the information of the NLC was already filtered out or not attended to. Thus, the increasing amount of LC was not competing with the information conveyed by the NLC. Our very high conflict faces were therefore not ambiguous.

This conjecture brings us to the second point needing explanations, *i*.*e*. why faces with 1 LC and sometimes also with 2 LC would be perceived as the most ambiguous faces and would result in longest response latencies? As proposed in the previous paragraph, NLC seemingly underwent no or only minor treatment. As also noted above, liars are likely to actively disguise their lying resulting in only some few lying indicators leaking [25]. From this we can suggest that faces with 1 LC (or 2 LC) are probably those that raise suspicion, *i*.*e*. are those being perceived as most ambiguous. This suspicion may lead from a modular to a higher cognitive processing mode. Such a switch in processing mode may imply that automatic and fast responses are hampered leading to more random unsure responses and higher reaction times for these decisions. Admittedly, our explanations are hypothetical; they raise more questions than having provided answers. Yet, comparable decision taking behavior in both studies is promising in pointing to replicable perception and decision-making processes in our paradigm. To better understand if a modular or controlled cognitive mode contributed to our findings, future studies are essential.

We have argued that reaction times would be a good behavioral parameter to decide whether a process is modular or not, because crucial characteristics of a modular system are its fastness and immediacy [5,15,16]. This assumption would indicate that increased reaction times together with random responding represent a non-modular processing style. This assumption would, however, also imply that we have some knowledge on cut-off scores for reaction times that distinguish between a modular mode and a non-modular mode of processing. Given that such cut-off scores are not available, our interpretation would require evidence from additional studies. For example, one such additional study could add cognitive load to the paradigm used here, *i*.*e*. requiring participants to perform a cognitive task before faces are presented [15,16]. Using such a paradigm, researchers could compare lying judgments when participants had performed a cognitive task just prior to the presentation of low or high conflicting faces. We would expect that such a cognitive load would be irrelevant to the processing of low conflicting faces [the modular system is not affected], but would interfere with the processing of high conflicting faces. In the later case, we could expect that (1) response latencies are even further enhanced if the processing mode is non-modular or (2) response latencies are reduced because the non-modular system is being occupied forcing the overall processing mode into a modular processing style. The answers to these possibilities could be pertinent; for instance when witnesses are interviewed in public or under psychological or physical pressure.

Another example for future studies concerns our suggestion regarding our first discussion point. We conjectured that NLC were not attended to or had already been filtered out. The reason for this irrelevance of NLC could be that they are as such not threatening or important for survival. Attending to lying information and ignoring non-lying information might be the default mode [5,11]. On the other hand, this processing bias may result from contextual information, *i*.*e*. the instruction. People were asked whether the faces are lying or not. Other interpersonal information was not mentioned. Our participants might have treated our faces differently if they would have had to decide whether the faces are trustworthy or not. Here, filtering and attention mechanisms might favor the processing of trustworthy cues. Also, we might find similar findings on trustworthiness if we would repeat the same studies again, but select faces according to trustworthiness.

Last but not least, we would like to stress the limitations of our studies. From a methodological point of view, we collected data online making it impossible to control for individual testing situations. Yet, previous studies showed that results from online data collection studies are comparable to those collected in a laboratory setting [37,38]. Also, during the determination of LC and NLC, we did not specify the characteristics of NLC. We only asked whether a face is lying or not. Potentially, more carefully selected NLC [*e*.*g*. trustworthy, honest, non-lying, etc.] would have been more relevant to the decision-making processes when being presented with increasingly conflicting faces. Moreover, one could question the face validity of our faces; these were static faces and were unrelated to real lies. Indeed, the literature on lying cues indicates that many different social cues relate to human expressions of lies such as body posture and voice (see, [4]). Using stimuli that control for more than facial features in static faces (see also, [18]), potentially even animated videos of people actually knowing to lie or not, would add important ecological validity to studies such as ours [5, 11, 15]. The challenge is to create controlled stimuli of increasing conflict. Indeed, a general problem inherent to the current study and related studies using artificial lying cues is that there is no real lying history attached to these stimuli. It may be that our results only reflect participants’ social impressions of others instead of their lying detection ability (see also [21]). One would like to know if our findings replicate for faces that were lying at the moment of being recorded. Finally, future studies should a priori account for possible differences in interpersonal judgements as a function of participants’ sex and culture [27,39,40].

## References

[1] Antonakis J, Dalgas O. Predicting elections: child’s play! Science. 2009; 323: 1183–1183. 10.1126/science.1167748 19251621

[2] Todorov A, Pakrashi M, Oosterhof NN. Evaluating faces on trustworthiness after minimal time exposure. Social Cognition. 2009; 27: 813–33.

[3] Ambady N, Bernieri FJ, Richeson JA. Toward a histology of social behavior: Judgmental accuracy from thin slices of the behavioral stream Advances in Experimental Social Psychology. Zanna Mark P.. San Diego, CA: Academic Press; 2000 p. 201–71.

[4] DePaulo BM, Lindsay JJ, Malone BE, Muhlenbruck L, Charlton K, Cooper H. Cues to deception. Psychological Bulletin. 2003; 129: 74–118. 12555795 10.1037/0033-2909.129.1.74

[5] Verplaetse J, Vanneste S, Braeckman J. You can judge a book by its cover: the sequel: A kernel of truth in predictive cheating detection. Evolution and Human Behavior. 2007; 28: 260–271.

[6] Schaller M. Evolutionary bases of first impressions In: Ambady N, Skowronski J., editors. First impressions. New York: Guilford Press; 2008 p. 15–34.

[7] Trivers RL. The evolution of reciprocal altruism. The quarterly review of biology. 1971; 46: 35–57.

[8] Sell A, Hagen EH, Cosmides L, Tooby J. Evolutionary psychology: applications and criticisms Encyclopedia of cognitive science. London: Macmillan; 2003 p. 47–53.

[9] Cosmides L, Tooby J. Neurocognitive adaptations designed for social exchange In: Buss DM, editor. The handbook of evolutionary psychology. Hoboken, NJ: Wiley; 2005 p. 584–627.

[10] Cosmides L, Tooby J. Cognitive adaptations for social exchange In: Barkow J, Cosmides L, Tooby J, editors. The adapted mind: Evolutionnary psychology and the generation of culture. New york: Oxford University Press; 1992 p. 163–228.

[11] Vanneste S, Verplaetse J, Vanhiel A, Braeckman J. Attention bias toward noncooperative people. A dot probe classification study in cheating detection. Evolution and Human Behavior. 2007; 28: 272–276.

[12] Chiappe D, Brown A, Dow B, Koontz J, Rodriguez M, McCulloch K. Cheaters are looked at longer and remembered better than cooperators in Social exchange situations. Evolutionary Psychology. 2004; 2: 108–120.

[13] Schupp HT, Öhman A, Junghöfer M, Weike AI, Stockburger J, Hamm AO. The facilitated processing of threatening faces: An ERP Analysis. Emotion. 2004; 4: 189–200. 15222855 10.1037/1528-3542.4.2.189

[14] Fodor JA. The modularity of mind: An essay on faculty psychology Cambridge, MA: MIT press; 1983.

[15] Bonnefon J-F, Hopfensitz A, De Neys W. The modular nature of trustworthiness detection. Journal of Experimental Psychology: General. 2013; 142: 143–50. 22686638 10.1037/a0028930

[16] Van Lier J, Revlin R, De Neys W. Detecting cheaters without thinking: testing the automaticity of the cheater detection module. Zalla T, editor. PLoS ONE. 2013; 8: e53827 10.1371/journal.pone.0053827 23342012 PMC3547066

[17] Manson JH, Gervais MM, Kline MA. Defectors cannot be detected during “small talk” with strangers. PloS one. 2013; 8: e82531 10.1371/journal.pone.0082531 24358201 PMC3865023

[18] Bond CF, DePaulo BM. Accuracy of deception judgments. Personality and social psychology Review. 2006; 10: 214–234. 16859438 10.1207/s15327957pspr1003_2

[19] Frank RH, Gilovich T, Regan DT. The evolution of one-shot cooperation: An experiment. Ethology and Sociobiology. 1993; 14: 247–256.

[20] Ekman P, O’ Sullivan M, Frank M. A few catch a liar. Psychological science. 1999; 10: 263–266.

[21] De Neys W, Hopfensitz A, Bonnefon J-F. Adolescents gradually improve at detecting trustworthiness from the facial features of unknown adults. Journal of Economic Psychology. 2015; 47: 17–22.

[22] Ekman P, O’Sullivan M, Friesen WV, Scherer KR. Invited article: Face, voice, and body in detecting deceit. Journal of nonverbal behavior. 1991; 15: 125–135.

[23] von Hippel W, Trivers R. The evolution and psychology of self-deception. Behavioral and Brain Sciences. 2011; 34: 1–16. 10.1017/S0140525X10001354 21288379

[24] Cheng PW, Holyoak KJ. On the natural selection of reasoning theories. Cognition. 1989; 33: 285–313. 2605884 10.1016/0010-0277(89)90031-0

[25] Buller DB, Burgoon JK. Interpersonal deception theory. Communication Theory. 1996; 6: 203–42.

[26] Okubo M, Kobayashi A, Ishikawa K. A Fake Smile thwarts cheater detection. Journal of Nonverbal Behavior. 2012; 36: 217–225.

[27] De Neys W, Hopfensitz A, Bonnefon J-F. Low second-to-fourth digit ratio predicts indiscriminate social suspicion, not improved trustworthiness detection. Biology Letters. 2013; 9: 143–150. 10.1098/rsbl.2013.0037 PMC3639781 23445949

[28] Little AC, Jones BC, DeBruine LM, Dunbar RIM. Accuracy in discrimination of self-reported cooperators using static facial information. Personality and Individual Differences. 2013; 54: 507–512.

[29] World Medical Association. World medical association declaration of helsinki: ethical principles for medical research involving human subjects. JAMA: The Journal of the American Medical Association. 310: 2191–2194. 10.1001/jama.2013.281053 24141714

[30] Manderscheid LV. Significance Levels. 0.05, 0.01, or? Journal of Farm Economics. 1965; 47: 1381–1385.

[31] Armstrong RA. When to use the Bonferroni correction. Ophthalmic and Physiological Optics. 2014; 34: 502–508. 10.1111/opo.12131 24697967

[32] Cabin RJ, Mitchell RJ. To Bonferroni or not to Bonferroni: when and how are the questions. Bulletin of the Ecological Society of America. 2000; 81: 246–248.

[33] Nakagawa S. A farewell to Bonferroni: the problems of low statistical power and publication bias. Behavioral Ecology. 2004; 15: 1044–1045.

[34] Rice WR. Analyzing tables of statistical tests. Evolution. 1989; 43: 223–225. 28568501 10.1111/j.1558-5646.1989.tb04220.x

[35] Norman DA, Bobrow DG. On data-limited and resource-limited Processes. Cognitive Psychology. 1975; 7: 44–64.

[36] Paas F, Tuovinen JE, Tabbers H, Van Gerven PW. Cognitive load measurement as a means to advance cognitive load theory. Educational psychologist. 2003; 38: 63–71.

[37] Skitka LJ, Sargis EG. The internet as psychological laboratory. Annual Review of Psychology. 2006; 57: 529–555. 16318606 10.1146/annurev.psych.57.102904.190048

[38] Lewis I, Watson B, White KM. Internet versus paper-and-pencil survey methods in psychological experiments: Equivalence testing of participant responses to health-related messages. Australian Journal of Psychology. 2009; 61: 107–116.

[39] Tognetti A, Berticat C, Raymond M, Faurie C. Is cooperativeness readable in static facial features? An inter-cultural approach. Evolution and Human Behavior. 2013; 34: 427–432.

[40] Kovács-Bálint Z, Bereczkei T, Hernádi I. The telltale face: Possible mechanisms behind defector and cooperator recognition revealed by emotional facial expression metrics. British Journal of Psychology. 2012; 104: 563–576. 10.1111/bjop.12007 24094284

